# Round the Hospitals

**Published:** 1917-04-07

**Authors:** 


### ROUND THE HOSPITALS.

It is with great regret that we have to report that Sister Grice while on duty in her ward at the Dunkirk Hospital was attacked by a patient and received serious injuries, from which we are glad to say she is making an excellent recovery. It has probably been noticed that Sister Grice, of the Queen Alexandra Hospital, Dunkirk, on March 17 was decorated with the Croix de Guerre by General Nollett, Commandant of the 36th Army Corps. Owing to the recent bombardments Sister Grice has been brought home earlier than would otherwise have been the case. We are sure the sympathy of the members of the nursing profession, here and in the United States, as well as in France, will go out to her in the circumstances of her injuries, from which we hope a complete recovery may speedily result.

A sister's bravery at a fire which occurred at Clevedon Red Cross Hospital, Somerset, on March 24 is likely to be heard of again. The matron in her report to headquarters dwells on the courage and coolness displayed by Sister Gladys Westrope, who was on night duty. She displayed a promptitude and ability in a most difficult situation which nothing could exceed. Alone she carried a helpless patient to safety and did everything possible to help. We are glad to be in a position to record that the matron, Miss Waddell, and her whole staff acted in a most exemplary and heroic manner on the occasion of the fire.

An experienced matron who has done splendid work in Poor-Law infirmaries makes a suggestion in regard to the many quite young women who are at present only half trained. She desires an opportunity to advise them to begin again. "Just now they would have a chance of getting on the staff of quite good hospitals, thus obtaining a proper training and a valuable certificate." We shall be glad to have the views of matrons and sisters generally upon this suggestion, and also to hear of any nurses who have experienced the difficulties referred to by our correspondent.

The *Poor-Law Officers' Journal* devotes much space to the article on "The Workhouse Influence" which we published in our issue of March 24, page 501. In referring to the Poor-Law nurse's manner to her patients, the writer in the *Journal* by his remarks illustrates very well the confusion in the public mind, and still more in the Poor-Law mind, between the well-trained infirmary nurse and the young woman employed in workhouse sick wards. The trained nurse is like a hospital nurse; she has the virtues and the failings of her profession, but is free from the workhouse tone with regard to her patients. This freedom she owes to the ceaseless vigilance of the superintendent of nurses and ward sisters, who nip it in the bud in the probationer's first year. A superintendent of nurses of the first rank in the Poor-Law Service has often been cheered by an experienced visitor's complimentary comment, "What nicely spoken girls your nurses are! They do not seem to have the workhouse manner at all." The former manner is a just source of pride to matrons of up-to-date Poor-Law infirmaries, for it is in striking contrast to the manner of nurses in the smaller Poor-Law establishments, where the opposite influence is so strong that the superintendent nurse there has had to give up fighting against it as "a bad job."

It would be well if the attention of Lord Rhondda, the President of the Local Government Board, could be directed to the difficulties created in the Poor-Law Nursing Service by the want of complete authority vested in the superintendent nurse in establishments where nurse-probationers are taken for training. The question of class in the Poor Law is of the first importance. Thus, we are assured, the probationer who goes into a general hospital and her sister into a large infirmary find themselves looked upon quite differently by visitors to the hospital and acquaintances at home, even their own friends. The loss of caste is very hard to bear, especially for girls of the lower middle class. Gentlewomen can afford to laugh at it, though it is sometimes a bitter joke even for them. The truth is the adequate facilities for nurse-training which should be afforded in every training-school under the Poor Law are frequently absent because the Local Government Board has not yet enforced the separation of Poor-Law sick accommodation where facilities can be given for the training of probationers free of control or interference from or by the workhouse master and the matron of the workhouse. If this step were taken it would be of great value to the nation, because then the status of the Poor-Law nurse would be raised to a standard equal to that enjoyed by those who have received their training and service in the best general hospitals up and down the country.

It is significant that the military authorities are requesting all auxiliary and V.A.D. hospitals under the Joint War Committee to increase their beds.

